# A Training Program for Professionals to Encourage Independence of Home-Living Older Adults: A Process Evaluation

**DOI:** 10.1093/geroni/igaa057.2150

**Published:** 2020-12-16

**Authors:** Teuni Rooijackers, G A Zijlstra, Erik van Rossum, Ruth G Vogel, Marja Veenstra, Gertrudis I J Kempen, Silke Metzelthin

**Affiliations:** 1 Maastricht University, Maastricht, Limburg, Netherlands; 2 Zuyd Hogeschool, Heerlen, Limburg, Netherlands; 3 Burgerkracht Libmurg, Sittard, Limburg, Netherlands

## Abstract

Stay Active at Home (SAaH) was developed to change homecare professionals’ behavior towards encouraging older adults’ independence in daily activities. This mixed-methods study evaluated SAaH regarding implementation, mechanisms of impact, and context. SAaH was implemented in five Dutch homecare teams (162 professionals). Quantitative data were collected from all professionals, and five focus groups with 23 professionals and 4 interventionists were performed. Data were analyzed using descriptive statistics and qualitative content analysis. SAaH was feasible to implement. Professionals visited on average 73% of the programme meetings. They reported positive changes in their knowledge, attitude, and skills, and perceived social and organizational support regarding the new way of working. The extent to which professionals applied SAaH in practice varied. SAaH was easier to apply among new clients. Perceived barriers were time pressure and staff shortages, and people’s resistance to change. Tailoring the intervention to professionals’ needs and wishes could improve their compliance. Part of a symposium sponsored by Nursing Care of Older Adults Interest Group.

